# Hydrogen Sulfide Regulates Salt Tolerance in Rice by Maintaining Na^+^/K^+^ Balance, Mineral Homeostasis and Oxidative Metabolism Under Excessive Salt Stress

**DOI:** 10.3389/fpls.2015.01055

**Published:** 2015-12-21

**Authors:** Mohammad G. Mostofa, Daisuke Saegusa, Masayuki Fujita, Lam-Son Phan Tran

**Affiliations:** ^1^Department of Biochemistry and Molecular Biology, Bangabandhu Sheikh Mujibur Rahman Agricultural UniversityGazipur, Bangladesh; ^2^Laboratory of Plant Stress Responses, Department of Applied Biological Science, Faculty of Agriculture, Kagawa UniversityMiki, Japan; ^3^Plant Abiotic Stress Research Group & Faculty of Applied Sciences, Ton Duc Thang UniversityHo Chi Minh City, Vietnam; ^4^Signaling Pathway Research Unit, RIKEN Center for Sustainable Resource ScienceYokohama, Japan

**Keywords:** global salinity, gasotransmitter, hydrogen sulfide, ionic stress, physiological and biochemical mechanisms, reactive oxygen species, rice, salt tolerance

## Abstract

Being a salt sensitive crop, rice growth and development are frequently affected by soil salinity. Hydrogen sulfide (H_2_S) has been recently explored as an important priming agent regulating diverse physiological processes of plant growth and development. Despite its enormous prospects in plant systems, the role of H_2_S in plant stress tolerance is still elusive. Here, a combined pharmacological, physiological and biochemical approach was executed aiming to examine the possible mechanism of H_2_S in enhancement of rice salt stress tolerance. We showed that pretreating rice plants with H_2_S donor sodium bisulfide (NaHS) clearly improved, but application of H_2_S scavenger hypotaurine with NaHS decreased growth and biomass-related parameters under salt stress. NaHS-pretreated salt-stressed plants exhibited increased chlorophyll, carotenoid and soluble protein contents, as well as suppressed accumulation of reactive oxygen species (ROS), contributing to oxidative damage protection. The protective mechanism of H_2_S against oxidative stress was correlated with the elevated levels of ascorbic acid, glutathione, redox states, and the enhanced activities of ROS- and methylglyoxal-detoxifying enzymes. Notably, the ability to decrease the uptake of Na^+^ and the Na^+^/K^+^ ratio, as well as to balance mineral contents indicated a role of H_2_S in ion homeostasis under salt stress. Altogether, our results highlight that modulation of the level of endogenous H_2_S genetically or exogenously could be employed to attain better growth and development of rice, and perhaps other crops, under salt stress. Furthermore, our study reveals the importance of the implication of gasotransmitters like H_2_S for the management of salt stress, thus assisting rice plants to adapt to adverse environmental changes.

## Introduction

Plants, being sessile in nature, are constantly exposed to an array of abiotic stresses throughout their life cycles. Salinity is one of the most brutal environmental constraints, affecting plant growth, development and productivity, especially in the arid and semi-arid regions (Parihar et al., 2015). High soil salinity has arisen as an important global concern, which hampers sustainable rice production in many agrarian countries like Bangladesh. Moreover, water shortage, hot and dry climate, and rising sea level due to global warming are aggravating the existing salinity problems, and thus worsening rice production in inland as well as coastal areas (Rabbani et al., 2013; Islam et al., 2015). Efforts to improve rice tolerance to salt stress through classical breeding have produced limited success and most of the existing varieties are unable to endure high salinity (Rabbani et al., 2013). Therefore, understanding the salt tolerance mechanisms of rice is indispensable in developing high salt-tolerant rice varieties to assure sustainable rice production in salt-affected regions.

Excessive salinity can induce various negative effects in plant cells, including ionic stress by accumulating Na^+^ and Cl^-^, osmotic stress by provoking water deficit, and oxidative stress by overproducing reactive oxygen species (ROS) (Munns and Tester, 2008; Gupta and Huang, 2014). Moreover, salinity can cause nutritional imbalance, especially K^+^ deficiency by inhibiting its uptake and triggering its leakage from the cells (Cuin et al., 2008; Munns and Tester, 2008; Parihar et al., 2015). Like other abiotic stresses, salinity also accelerates the generation of methylglyoxal (MG), a highly cytotoxic compound, which can crosslink with DNA, RNA, and proteins, thereby exacerbating oxidative stress (Yadav et al., 2005; Saito et al., 2011; Hossain et al., 2012).

Salt tolerance is a complex trait that includes a variety of defense mechanisms, including ionic, osmotic and ROS homeostases (Miller et al., 2010; Gupta and Huang, 2014). Plants respond to ionic imbalance by regulating ion fluxes and mineral homeostases. Several plant species have the potential to defend ionic and osmotic stresses by producing compatible solutes, such as proline and trehalose, to maintain water relations, stabilize enzyme and protein complexes and membranes under high salinity conditions (Mostofa et al., 2014b; Iqbal et al., 2015; Reddy et al., 2015). Plant protection against salt-induced ROS is closely related to the maintenance of cellular redox balance, which is mainly composed of several non-enzymatic antioxidants, such as ascorbic acid (AsA) and glutathione (GSH), and enzymatic antioxidants, such as superoxide dismutase (SOD), catalase (CAT), glutathione peroxidase (GPX), glutathione *S*-transferase (GST), ascorbate peroxidase (APX), monodehydroascorbate reductase (MDHAR), dehydroascorbate reductase (DHAR), and glutathione reductase (GR) (Shao et al., 2007; Gill and Tuteja, 2010; Miller et al., 2010). Likewise, plant cells also possess a GSH-based glyoxalase (Gly) system that detoxifies reactive MG by consecutive actions of Gly I and Gly II (Hossain et al., 2012). Transgenic studies revealed that the antioxidant defense and glyoxalase systems are intermingled, and efficient induction of these systems was correlated with enhanced tolerance to abiotic stresses in different crop varieties (Mustafiz et al., 2011; Upadhyaya et al., 2011; Alvarez Viveros et al., 2013).

Efforts are continuing to enhance plants’ capacity to survive under environmental stresses. In this context, execution of the potential roles of small biological molecules, such as phytohormones and signal molecules could be considered as a powerful tool in modifying plants’ adaptability against adverse effects resulted from ever-changing global conditions. Hydrogen sulfide (H_2_S), a small bioactive gas with rotten egg disodour, has emerged as a third gasotransmitter after nitric oxide and carbon monoxide in animal systems, and is implicated in numerous health benefits (Mancardi et al., 2009; Toit, 2015). In plant systems, H_2_S has recently come into light because of its association with adaptive responses against environmental stressors. Recent studies showed that priming of plants with H_2_S donor sodium bisulfide (NaHS) offered significant tolerance to a number of abiotic stresses, including drought, heat, heavy metals and osmotic stresses (Christou et al., 2013; Shi et al., 2013; Ali et al., 2014; Mostofa et al., 2015d). The positive effects of H_2_S in stress mitigation have been attributed to its association with multiple defense mechanisms, such as antioxidant activities and modulation of ROS detoxification system (Chen et al., 2011; Mostofa et al., 2015d). However, the underlying mechanisms H_2_S uses to regulate salt tolerance, especially in major crops like rice, are still elusive, which requires in-depth analysis at the physiological and biochemical levels.

Thus, in the current study, we aimed to evaluate the potential roles of H_2_S in addressing the problems associated with the detrimental consequences of global salinity in an economically important crop. For this purpose, we have examined the physiological and biochemical mechanisms associated with H_2_S-induced salt tolerance in rice, with particular emphasis on ROS- and MG-detoxifying systems. At the same time, the role of H_2_S in regulating Na^+^/K^+^ balance and mineral homeostases was also examined. Our results support the notion that H_2_S could serve as an important priming agent in enhancing rice tolerance to salt stress, particularly by inhibiting Na^+^ uptake, reestablishing redox and mineral homeostases, as well as reducing oxidative stress by stimulating ROS and MG detoxifications.

## Materials and Methods

### Plant Materials, Growth Conditions and Treatments

The germination and cultivation of rice (*Oryza sativa* L. cv. BRRI dhan52) in a hydroponic condition were carried out according to the methods described by Mostofa et al. (2014b, 2015a,b). After 12 days, healthy plants were pretreated with H_2_S for 48 h by supplying 0, 25, 50, 100, and 200 μM NaHS in the nutrient solution. Hypotaurine (HT), a scavenger of H_2_S (Christou et al., 2013), was applied only with 50 μM NaHS at a concentration of 500 μM to justify the H_2_S-dependent role of NaHS in salt tolerance. Pretreated and non-pretreated plants were exposed to nutrient solution containing 150 mM NaCl and further grown for 4 days in the specified conditions. This environmentally relevant NaCl concentration, which exhibited visible toxic symptoms on rice plants after 4 days of treatment, was selected based on our previous studies (Mostofa et al., 2014a, 2015a). The second leaf of rice seedlings was harvested to determine various physiological and biochemical responses altered by the application of NaHS, HT and NaCl. Each treatment was replicated three times under the same experimental conditions.

### Assessment of Growth Parameters

The growth of rice seedlings was assessed by measuring plant height, fresh weight (FW) and dry weight (DW). Plant height was determined by measuring the length from the bottom of the main stem to the end of the emerging third leaf. To determine FW and DW, 10 seedlings from each treatment were weighed (FW) and oven-dried at 80°C for 48 h (DW), then expressed as g seedling^-1^.

### Determination of H_2_S Content

H_2_S content in rice leaves was determined following the method described by Christou et al. (2013) with modifications. Rice leaves (0.25 g) were homogenized in 1 mL of 100 mM K-P buffer (pH 7.0) containing 10 mM EDTA, then centrifuged at 11,200 × *g* for 15 min. The supernatant (100 μL) was mixed with 1,880 μL extraction buffer and 20 μL of 20 mM 5,5′-dithiobis (2-nitrobenzoic acid) and incubated at 25°C for 5 min. The absorbance was read at 412 nm, and H_2_S was quantified using a standard curve developed with known concentrations of NaHS.

### Measurement of Na, K and Mineral Nutrient Contents in Roots and Leaves

To determine Na, K and mineral nutrient contents (Ca, Mg, Fe, Zn, and Mn), the root and leaf samples were harvested separately and oven-dried at 80°C for 48 h. Dried samples (0.1 g) were ground and digested with a HNO_3_:HClO_4_ (5:1 v/v) mixture at 80°C until the yellow color disappeared. The Na, K, Ca, Mg, Fe, Zn, and Mn contents were measured by using flame atomic absorption spectrophotometry.

### Relative Water Content and Proline Content

Relative water content (RWC) was determined as described by Mostofa et al. (2015a). Proline (Pro) content was determined according to the method of Bates et al. (1973) with minor modifications as reported in Mostofa and Fujita (2013). Freshly collected leaves (0.5 g) were homogenized with 5 mL of 3% aqueous sulfosalicylic acid. After centrifugation at 11,500 × *g* for 15 min, the supernatant (1 mL) was mixed with 2 mL solution containing glacial acetic acid and acid ninhydrin (1:1, v/v). Subsequently, the mixture was boiled at 100°C for 1 h and then transferred to ice to stop the reaction. Toluene (2 mL) was used to extract the developed color of the chromophore and the absorption was measured at 520 nm. Pro content was determined using a calibration curve developed by Pro standard.

### Photosynthetic Pigments

After extracting 0.5 g leaves in 80% chilled acetone, the absorbance of the supernatants was recorded at 463, 445, and 470 nm for determining the contents of chlorophylls (Chl) and carotenoids according to the formulas suggested by Arnon (1949) and Lichtenthaler and Wellburn (1983), respectively.

### Lipid Peroxidation and H_2_O_2_ Content

Lipid peroxidation of the leaves was measured by estimating malondialdehyde (MDA) according to the method of Heath and Packer (1968). H_2_O_2_ was extracted and determined after reaction with 0.1% TiCl_4_ in 20% H_2_SO_4_ following the method of Mostofa and Fujita (2013).

### Histochemical Analyses

For observing the accumulation of ROS, namely $O_{2}^{\cdot -}$ and H_2_O_2_, in leaves, histochemical analyses were carried out in rice leaves following the method of Mostofa and Fujita (2013). Briefly, 0.1% nitroblue tetrazolium (NBT) or 1% 3, 3-diaminobenzidine (DAB) solution was used to stain the cut leaves under dark and light, respectively. After incubation for 24 h, incubated leaves were decolorized by immersing in boiling ethanol to visualize the blue insoluble formazan for $O_{2}^{\cdot -}$ or deep brown polymerization product for H_2_O_2_. After cooling, photographs of the stained leaves were taken by placing between two glass plates.

### Extraction and Estimation of Non-enzymatic Antioxidants

Fresh leaves (0.5 g) was homogenized in 3 mL of ice-cold 5% meta-phosphoric acid containing 1 mM EDTA and centrifuged at 11,500 × *g* for 15 min. Reduced and total AsA contents were assayed at 265 nm following the method of Mostofa et al. (2015b). Oxidized ascorbate (DHA; dehydroascorbic acid) content was determined by subtracting reduced AsA value from total AsA content. Total GSH and oxidized glutathione (GSSG) contents were calculated as described by Griffiths (1980). Reduced GSH content was measured after deducting the GSSG amount from total GSH level.

### Extraction and Assay of Enzymes

Extraction of enzymes was carried out following the method of Mostofa et al. (2015a,b). Activities of antioxidant and glyoxalase enzymes were determined by the standard methods reported in Doderer et al. (1992) for lipoxygenase (LOX, EC 1.13.11.12), Mostofa and Fujita (2013) for SOD (EC 1.15.1.1) and CAT (EC 1.11.1.6), Nakano and Asada (1981) for APX (EC 1.11.1.11) and DHAR (EC 1.8.5.1), Hossain et al. (1984) for MDHAR (EC 1.6.5.4), Mostofa and Fujita (2013) for GR (EC 1.6.4.2), GST (EC 2.5.1.18) and GPX (EC: 1.11.1.9), Hossain et al. (2009) for Gly I (EC 4.4.1.5) and Mostofa and Fujita (2013) for Gly II (EC 3.1.2.6), respectively. Protein content was determined following the method of Bradford (1976) using bovine serum albumin (BSA) as a standard.

### Estimation of Methylglyoxal (MG) Content

The MG content in rice leaves was determined following the method of Wild et al. (2012) as essentially described in Mostofa et al. (2015b) with exception that 0.5 g of leaves was used instead of 0.25 g.

### Statistical Analysis

The data were subjected to one-way analysis of variance (ANOVA) and different letters indicate significant differences among treatments at *p* < 0.05, according to Duncan’s multiple range test (DMRT) using IRRISTAT version 3 (IRRI, Biometrics Unit, Manila, Philippines). Data represented in the Tables and Figures are means ± standard deviations (SDs) of three replicates for each treatment.

## Results

### Effects of Exogenous NaHS on Endogenous Level of H_2_S and Selection of Effective Dose of NaHS

**FIGURE 1:**
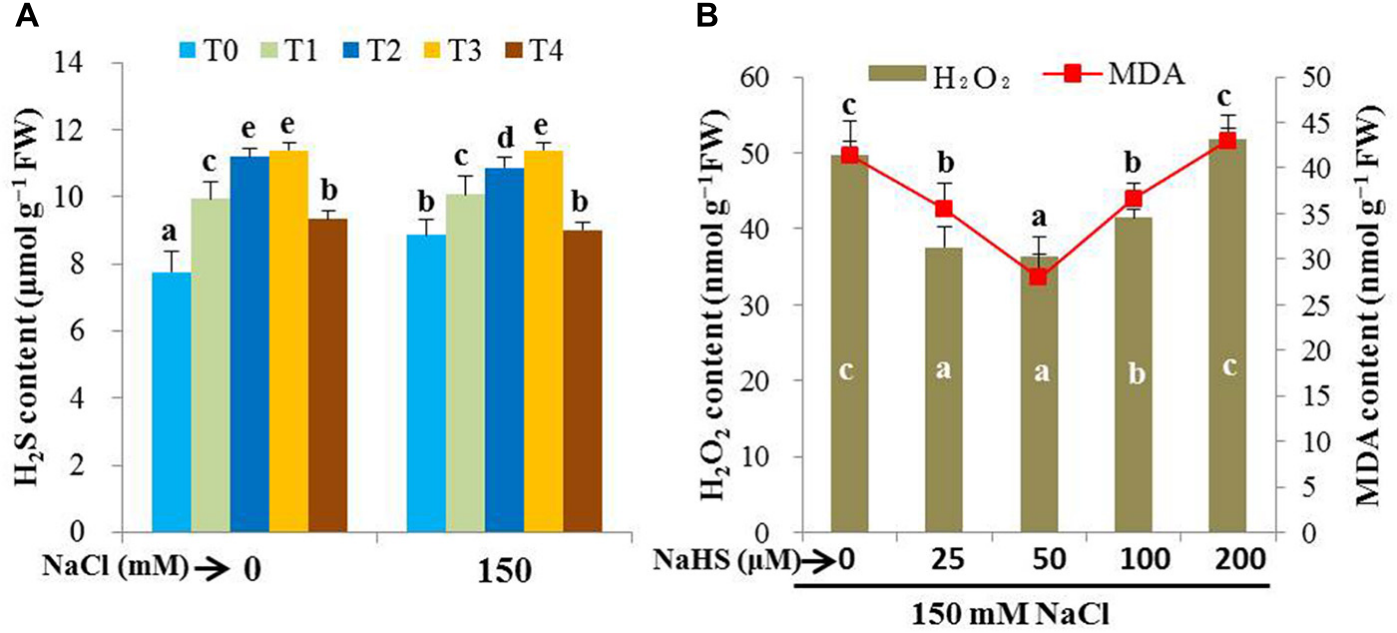
**Evaluation of effective dose of NaHS**. Effects of exogenous NaHS on the levels of endogenous H_2_S **(A)** and oxidative stress indicators, H_2_O_2_ and MDA **(B)** in the leaves of salt-stressed rice plants pretreated with or without varying doses of NaHS. T0, T1, T2, T3, and T4 indicate 0 μM NaHS, 25 μM NaHS, 50 μM NaHS, 100 μM NaHS and 50 μM NaHS + 500 μM HT, respectively. Bars represent standard deviation (SD) of the mean (*n* = 3). Different letters (a, b, c, d, and e) indicate significant differences among the treatments at *P <* 0.05, according to Duncan’s multiple range test. HT, hypotaurine.

The protective role of H_2_S was assessed by examining the effects of H_2_S donor NaHS on the level of endogenous H_2_S, and the levels of H_2_O_2_ and MDA in the leaves of rice plants under salt stress. Under non-stressed conditions, the pretreatment of rice plants with 25 (T1), 50 (T2) and 100 μM NaHS (T3) for 48 h increased the levels of H_2_S by 28, 44, and 47%, respectively, as compared with the non-pretreated control plants (**Figure 1A**). We also observed that the pretreated rice plants maintained a higher level of H_2_S when subjected to 150 mM NaCl stress for 4 days (14, 23 and 28%, respectively), as compared with salt-treated only plants. To clarify the role of H_2_S released from NaHS, we applied 500 μM HT with 50 μM NaHS (T4) under both normal and stress conditions and observed that HT addition reversed NaHS-induced increase in H_2_S level (**Figure 1A**). This result indicated the contribution of exogenous NaHS in elevating the level of endogenous H_2_S. To further confirm the effective dose of NaHS, oxidative stress indicators, such as H_2_O_2_ and MDA, were estimated in the plants treated with varying concentrations of NaHS prior to salt stress. The pretreatment with 50 μM NaHS was shown to be most effective in decreasing the levels of H_2_O_2_ and MDA in salt-stressed seedlings (**Figure 1B**). Interestingly, a further increase in the concentration of NaHS aggravated the levels of these stress indicators. Taken together, 50 μM NaHS and 500 μM HT were used to test the protective role of H_2_S against high salinity (150 mM) in the subsequent experiments.

### H_2_S Maintains Na^+^ and K^+^ Homeostases

**FIGURE 2:**
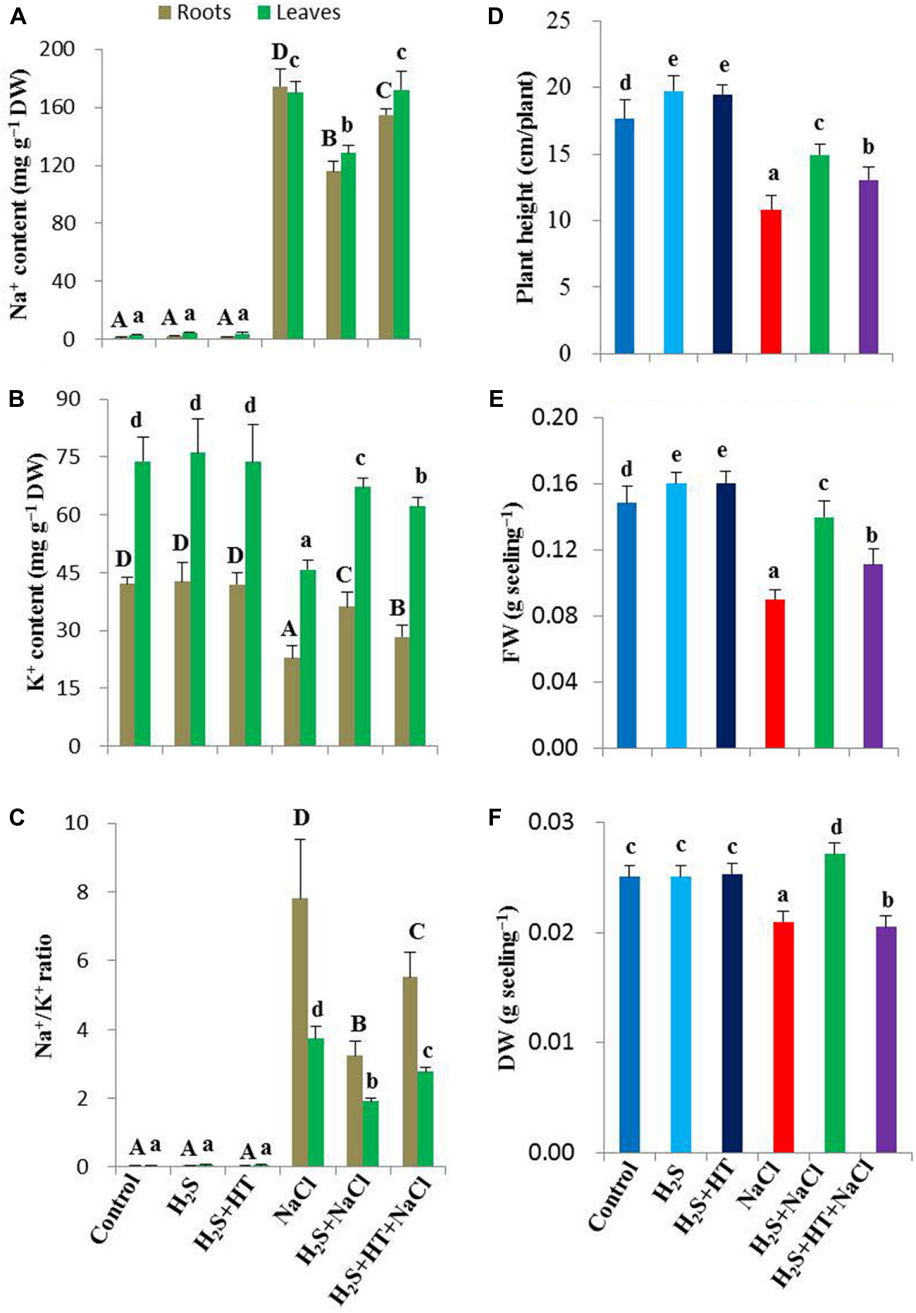
**Effects of H_2_S on Na^+^ and K^+^ status, growth and biomass of salt-stressed rice plants pretreated with or without NaHS and HT. (A)** Na^+^ content, **(B)** K^+^ content, **(C)** Na^+^/K^+^ ratio, **(D)** plant height, **(E)** fresh weight and **(F)** dry weight. Control, H_2_S, H_2_S + HT, NaCl, H_2_S + NaCl, H_2_S + HT + NaCl correspond to the group of seedlings subjected to only nutrients, 50 μM NaHS, 50 μM NaHS + 500 μM HT, 150 mM NaCl, 50 μM NaHS + 150 mM NaCl and 50 μM NaHS + 500 μM HT + 150 mM NaCl, respectively. Bars represent standard deviation (SD) of the mean (*n* = 3). Different letters (A, B, C, and D for roots and a, b, c, d, and e for leaves) indicate significant differences among the treatments at *P <* 0.05, according to Duncan’s multiple range test. DW, dry weight; HT, hypotaurine.

Maintaining Na^+^ and K^+^ homeostases is crucial for plant survival under salt stress conditions. Salt treatment resulted in a noticeable increase in Na^+^ content (15,069 and 5,515% in roots and leaves, respectively) and a significant decrease in K^+^ content (46 and 38% in roots and leaves, respectively) in salt-stressed plants compared with untreated control (**Figures 2A,B**). Accordingly, Na^+^/K^+^ ratio significantly increased in roots and leaves of salt-treated plants in comparison with untreated control plants. On the other hand, pretreatment with NaHS maintained Na^+^ and K^+^ homeostases, as Na^+^ content decreased by 34 and 25%, and K^+^ content increased by 58 and 47% in roots and leaves of salt-stressed plants, respectively, compared with salt-stressed only plants (**Figures 2A,B**). Consequently, Na^+^/K^+^ ratio was significantly lower in NaHS-pretreated salt-stressed plants when compared with the plants treated with salt alone (**Figure 2C**). However, adding both HT and NaHS disturbed Na^+^ and K^+^ homeostases, resulting in similar accumulation of Na^+^ and K^+^ in the salt-stressed plants pretreated with and without both HT and NaHS (**Figures 2A–C**), which indicated that scavenging of H_2_S by HT predisposed the salt-stressed plants to suffering from NaCl toxicity. Thus, H_2_S eases the life of rice plants under salt stress, notably by inhibiting excessive Na^+^ uptake and by maintaining K^+^ homeostasis at the cellular level.

### H_2_S Regulates Plant Growth and Biomass

To evaluate the effects of H_2_S on growth and biomass of rice plants under NaCl stress for 4 days, we determined the height, FW and DW of rice plants. Salt stress decreased the plant height by 39% in salt-stressed only seedlings compared with untreated control (**Figure 2D**). FW and DW also reduced by 40 and 16%, respectively, in salt-stressed seedlings when compared with control (**Figures 2E,F**). The pretreatment with NaHS; however, significantly increased the plant height, FW and DW in salt-stressed plants by 38, 78, and 29%, respectively, in comparison with salt-stressed only plants (**Figures 2D–F**). On the other hand, adding HT with NaHS during pretreatment negatively affected the plant growth and biomass recovery in salt-stressed plants. Under non-stress conditions, plant height and FW significantly increased, whereas DW remained constant in NaHS- and NaHS+HT-pretreated plants relative to control plants (**Figures 2D–F**).

### H_2_S Reestablishes Essential Mineral Homeostases

To provide an insight into the role of H_2_S in regulating mineral homeostases under salt stress, we estimated the levels of Ca, Mg, Fe, Zn, and Mn in both roots and leaves of rice plants (**Table 1**). In the roots of salt-stressed plants, Ca, Mg, Fe, Zn, and Mn contents decreased by 34, 29, 19, 22, and 14%, respectively, relative to the control plants. As for leaves, salt stress caused a decrease in the contents of Ca and Mg by 24 and 25%, respectively, whereas it resulted in an increase in Fe, Zn, and Mn contents by 50, 70, and 63%, respectively, over the control values. On the other hand, NaHS treatment prior to salt exposure significantly decreased the negative effects of high salinity on the contents of these minerals (**Table 1**). A lower decrease of Ca content in roots (6%) and leaves (7%) of NaHS-pretreated salt-stressed plants was observed in relation to control plants. In comparison with salt-stressed only plants, Mg, Fe, Zn, and Mn contents remained 50, 38, 76, and 75% higher in roots and 41, 29, 24, and 79% higher in leaves of NaHS-pretreated salt-stressed plants, respectively. Conversely, co-application of NaHS and HT prior to salt stress treatment disturbed the mineral homeostases in both roots and leaves, in which Ca, Mg, Fe, Zn, and Mn contents remained at the levels similar to those of salt-stressed only plants. Under non-stress conditions, addition of NaHS significantly improved the contents of these minerals in both roots and leaves compared with control (**Table 1**). Collectively, our results implied that NaHS pretreatment played a significant role in maintaining mineral homeostases in rice plants under both normal and salt stress conditions.

**Table 1:** Effects of H_2_S on the levels of essential minerals in the roots and leaves of rice plants with or without NaCl stress.

Treatment	Ca (mg g^-1^DW)		Mg (mg g^-1^DW)		Fe (mg g^-1^DW)		Zn (mg g^-1^DW)		Mn (mg g^-1^DW)	
	Roots	Leaves	Roots	Leaves	Roots	Leaves	Roots	Leaves	Roots	Leaves
Control	3.96 ± 0.10^c^	9.15 ± 1.12^c^	32.47 ± 5.18^c^	53.52 ± 5.26^c^	8.68 ± 0.66^b^	0.68 ± 0.03^a^	0.32 ± 0.03^b^	0.20 ± 0.02^a^	0.14 ± 0.02^c^	0.32 ± 0.04^a^
H_2_S	4.26 ± 0.05^d^	9.33 ± 0.95^c^	34.61 ± 0.42^d^	59.49 ± 3.45^e^	9.48 ± 1.81^c^	1.13 ± 0.21^c^	0.39 ± 0.03^c^	0.26 ± 0.02^b^	0.21 ± 0.03^e^	0.67 ± 0.05^c^
H_2_S + HT	3.80 ± 0.18^b^	8.18 ± 0.55^b^	35.43 ± 2.01^d^	57.08 ± 2.26^d^	9.92 ± 1.24^c^	1.19 ± 0.11^c^	0.35 ± 0.04^b^	0.26 ± 0.03^b^	0.17 ± 0.03^d^	0.79 ± 0.05^e^
NaCl	2.62 ± 0.16^a^	6.94 ± 0.47^a^	23.07 ± 1.14^a^	39.94 ± 1.61^a^	7.07 ± 0.52^a^	1.02 ± 0.10^b^	0.25 ± 0.03^a^	0.34 ± 0.04^d^	0.12 ± 0.02^b^	0.52 ± 0.06^b^
H_2_S + NaCl	3.73 ± 0.12^b^	8.51 ± 0.53^b^	34.50 ± 4.01^d^	56.50 ± 2.01^d^	9.79 ± 1.65^c^	1.32 ± 0.06^d^	0.44 ± 0.06^d^	0.42 ± 0.02^e^	0.21 ± 0.02^e^	0.93 ± 0.03^f^
H_2_S + HT + NaCl	2.55 ± 0.22^a^	6.78 ± 0.47^a^	26.29 ± 3.14^b^	49.75 ± 3.35^b^	8.36 ± 0.57^b^	1.16 ± 0.11^c^	0.33 ± 0.02^b^	0.31 ± 0.07^c^	0.09 ± 0.07^a^	0.72 ± 0.09^d^

*Control, H_*2*_S, H_*2*_S + HT, NaCl, H_*2*_S + NaCl, H_2_S + HT + NaCl correspond to the group of seedlings exposed to only nutrients, 50 μM NaHS, 50 μM NaHS + 500 μM hypotaurine, 150 mM NaCl, 50 μM NaHS + 150 mM NaCl and 50 μM NaHS + 500 μM hypotaurine + 150 mM NaCl, respectively. FW, fresh weight. Values are means ± SD of three independent replications (*n* = 3). Different superscripted letters (a, b, c, d, e, and f) within the column indicate statistically significant differences among the treatments according to Duncan’s multiple range test (*P <* 0.05)*.

### H_2_S Suppresses ROS Accumulation

**FIGURE 3:**
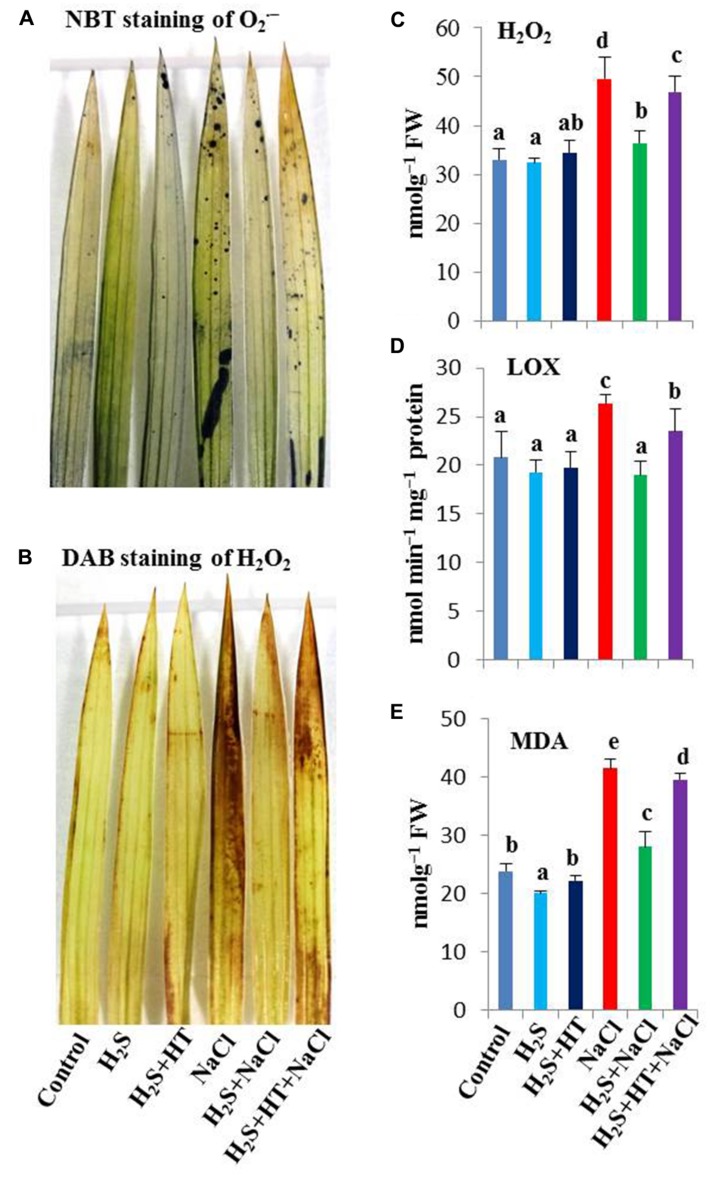
**Effect of H_2_S on oxidative stress indices in the leaves of salt-stressed rice plants pretreated with or without NaHS and HT. (A)** Dark blue spots indicated accumulation of superoxide ( $O_{2}^{\cdot -}$), **(B)** brown polymerization products indicated accumulation of hydrogen peroxide (H_2_O_2_), **(C)** H_2_O_2_ content, **(D)** lipoxygenase (LOX) activity and **(E)** malondialdehyde (MDA) content. Control, H_2_S, H_2_S + HT, NaCl, H_2_S + NaCl, H_2_S + HT + NaCl correspond to the group of seedlings subjected to only nutrients, 50 μM NaHS, 50 μM NaHS + 500 μM HT, 150 mM NaCl, 50 μM NaHS + 150 mM NaCl and 50 μM NaHS + 500 μM HT + 150 mM NaCl, respectively. Bars represent standard deviation (SD) of the mean (*n* = 3). Different letters (a, b, c, d, and e) indicate significant differences among the treatments at *P <* 0.05, according to Duncan’s multiple range test. FW, fresh weight; HT, hypotaurine; NBT, nitroblue tetrazolium; DAB, diaminobenzidine.

To clarify the protective role of H_2_S against NaCl-induced oxidative stress, we carried out histochemical detection of major ROS like $O_{2}^{\cdot -}$ and H_2_O_2_ in the leaves of rice plants with or without salt stress. As shown in **Figures 3A,B** the rice plants treated with NaCl alone showed extensive staining with NBT as dark blue spots (for $O_{2}^{\cdot -}$) and DAB as brown polymerization products (for H_2_O_2_), while those pretreated with NaHS had only slight staining. Pretreatment with both NaHS and HT exacerbated the accumulation of $O_{2}^{\cdot -}$ and H_2_O_2_, showing similar staining intensity to that of salt-stressed only seedlings (**Figures 3A,B**). This result suggested that enhanced H_2_S level conferred ROS homeostasis, thereby protecting rice plants from NaCl-induced oxidative stress.

### H_2_S Reduces Oxidative Stress

Reactive oxygen species, such as H_2_O_2_, and lipid peroxidation products, such as MDA, are recognized as cellular indicators of oxidative stress (Miller et al., 2010). Salt stress caused a dramatic increase of H_2_O_2_ by 50% in comparison with untreated control (**Figure 3C**). LOX, as an oxidative enzyme, often participates in lipid peroxidation contributing to oxidative stress (Kumar et al., 2013). We observed that salt stress increased the activity of LOX by 26% compared with control (**Figure 3D**). As a consequence, MDA content increased by 74% in salt-stressed plants over control plants that coincided with the higher H_2_O_2_ level and increased LOX activity. However, treating rice plants with NaHS prior to salt treatment contributed to the reduction of H_2_O_2_ (27%), LOX activity (28%) and subsequent MDA level (34%) compared with salt-stressed only plants (**Figure 3E**). In contrast, co-application of HT and NaHS during pretreatment inclined rice plants to suffer from higher oxidative load. In these plants, H_2_O_2_ content, LOX activity and MDA content remained significantly higher than that of NaHS-pretreated salt-exposed plants (**Figures 3C–E**), indicating that H_2_S effectively protected rice plants from oxidative stress. Compared with untreated control, NaHS and NaHS + HT pretreatments alone did not significantly affect the levels of H_2_O_2_ and MDA as well as the LOX activity.

### H_2_S Protects Photosynthesis Pigments, Water Soluble Proteins and Water Status

In comparison with control, a sharp decline in the content of Chl a (18%), Chl b (31%), total Chl (21%), carotenoids (16%) and water soluble proteins (20%) was recorded in the leaves of salt-stressed rice plants (**Table 2**). Applying NaHS prior to salt stress treatment significantly improved the contents of these parameters. The levels of Chl a, Chl b, total Chl, carotenoids and soluble proteins were 22, 46, 33, 22, and 33%, respectively, higher in the leaves of NaHS-pretreated salt-stressed plants than in that of salt-stressed only plants (**Table 2**). Nevertheless, the H_2_S-mediated recovery on the losses of photosynthetic pigments and soluble protein contents was significantly reduced due to simultaneous application of NaHS and HT prior to salt treatment, when compared with NaHS pretreatment alone (**Table 2**). Under non-stress conditions, NaHS did not alter the levels of photosynthetic pigments and water soluble proteins but co-pretreatment of NaHS and HT significantly reduced the levels of Chl b and water soluble proteins compared with control.

**Table 2:** Effects of H_2_S on the levels of chlorophyll (Chl) a, b, total Chl, carotenoids, water soluble proteins, relative water content (RWC) and proline (Pro) in the leaves of rice plants with or without NaCl stress.

Treatment	Chl a (mg g^-1^ FW)	Chl b (mg g^-1^ FW)	Chl (a + b) (mg g^-1^ FW)	Carotenoids (mg g^-1^ FW)	Water soluble proteins (mg g^-1^ FW)	RWC (%)	Pro (μmol g^-1^ FW)
Control	2.68 ± 0.05^c^	0.81 ± 0.06^d^	3.49 ± 0.11^c^	0.70 ± 0.02^c^	17.49 ± 0.05^c^	98.85 ± 0.89^e^	0.15 ± 0.02^a^
H_2_S	2.70 ± 0.06^c^	0.80 ± 0.07^d^	3.50 ± 0.06^c^	0.71 ± 0.01^c^	17.53 ± 0.47^c^	97.79 ± 1.53^de^	0.15 ± 0.04^a^
H_2_S + HT	2.72 ± 0.06^c^	0.74 ± 0.04^c^	3.51 ± 0.06^c^	0.68 ± 0.01^c^	16.57 ± 0.24^b^	96.93 ± 1.99^d^	0.20 ± 0.02^a^
NaCl	2.20 ± 0.10^a^	0.56 ± 0.04^a^	2.75 ± 0.07^a^	0.58 ± 0.02^a^	13.98 ± 0.74^a^	74.18 ± 1.33^a^	4.93 ± 0.20^d^
H_2_S + NaCl	2.83 ± 0.04^d^	0.82 ± 0.06^d^	3.65 ± 0.05^d^	0.71 ± 0.02^c^	18.57 ± 0.41^d^	88.20 ± 1.69^c^	3.10 ± 0.61^b^
H_2_S + HT + NaCl	2.39 ± 0.16^b^	0.61 ± 0.05^b^	3.00 ± 0.18^b^	0.66 ± 0.05^b^	16.35 ± 0.11^b^	77.40 ± 1.14^b^	3.98 ± 0.57^c^

*Control, H_*2*_S, H_*2*_S + HT, NaCl, H_*2*_S + NaCl, H_2_S + HT + NaCl correspond to the group of seedlings exposed to only nutrients, 50 μM NaHS, 50 μM NaHS + 500 μM hypotaurine, 150 mM NaCl, 50 μM NaHS + 150 mM NaCl and 50 μM NaHS + 500 μM hypotaurine + 150 mM NaCl, respectively. FW, fresh weight. Values are means ± SD of three independent replications (*n* = 3). Different superscripted letters (a, b, c, d, and e) within the column indicate statistically significant differences among the treatments according to Duncan’s multiple range test (*P <* 0.05)*.

Leaf RWC decreased by 25% in salt-stressed only plants compared with the untreated control (**Table 2**). However, the observed 19% increase in leaf RWC in NaHS-pretreated salt-stressed plants relative to the plants stressed with salt alone indicated that exogenous NaHS significantly prevented the reduction of RWC in rice plants under salt stress. On the other hand, H_2_S-mediated restoration of leaf RWC significantly reversed when NaHS and HT were applied together (**Table 2**). Salt stress also resulted in a significant increase in Pro content (3,187%), whereas NaHS pretreatment showed lower increase in Pro content (1,967%) in the leaves of salt-stressed plants in comparison with untreated control plants (**Table 2**). Conversely, adding HT with NaHS during pretreatment significantly increased Pro level by 2,553% in salt-stressed plants compared with control plants. NaHS did not affect the leaf RWC and Pro level under non-stress conditions. However, co-pretreatment of NaHS and HT increased Pro content by 33% compared with control (**Table 2**).

### H_2_S Enhances the Levels of AsA and GSH, and Modulation of Redox States

Ascorbic acid and GSH, two vital water soluble non-enzymatic antioxidants, can detoxify ROS either directly or indirectly by helping enzymes involved in H_2_O_2_ metabolism (Shao et al., 2008; Gill and Tuteja, 2010). In parallel with ROS, we investigated the H_2_S-induced modulation of AsA, GSH and redox states in rice leaves under salt stress. With respect to the untreated control, salt stress led to a noticeable reduction of AsA content (39%) and increment of DHA content (17%), while a significant increase in GSH and GSSG contents by 43 and 189%, respectively (**Table 3**). This salt-induced modulation of AsA and GSH pools also led to decrease in AsA/DHA and GSH/GSSG ratios in the leaves of the plants treated with salt alone. However, NaHS treatment prior to salt stress significantly recovered salt-induced loss of AsA content as well as further enhanced the GSH content, leading to a maintenance in AsA/DHA and GSH/GSSG ratios (**Table 3**) that are important determinants of cell survival under stressful conditions. A contrasting change in AsA and GSH contents and the AsA/DHA and GSH/GSSG ratios was observed when HT was applied along with NaHS during pretreatment (**Table 3**). Thus, H_2_S-mediated elevation of these antioxidants and redox states might have contributed to ROS detoxification under salt stress.

**Table 3:** Effects of H_2_S on the levels of non-enzymatic antioxidants ascorbic acid (AsA) and glutathione (GSH), as well as their redox states (AsA/DHA and GSH/GSSG) in the leaves of rice plants with or without NaCl stress.

Treatment	AsA (nmol g^-1^ FW)	DHA (nmol g^-1^ FW)	AsA/DHA ratio	GSH (nmol g^-1^ FW)	GSSG (nmolg^-1^ FW)	GSH/GSSG ratio
Control	4194.19 ± 260.34^d^	792.58 ± 51.61^b^	5.32 ± 0.61^cd^	465.57 ± 13.02^a^	27.41 ± 2.78^a^	17.13 ± 2.17^c^
H_2_S	4287.10 ± 161.55^d^	773.23 ± 87.84^b^	5.83 ± 1.75^d^	506.60 ± 9.44^b^	29.88 ± 4.59^ab^	17.23 ± 2.71^c^
H_2_S+ HT	3827.42 ± 318.59^c^	778.06 ± 49.37^b^	5.06 ± 1.15^c^	475.04 ± 16.52^a^	39.10 ± 4.21^c^	12.25 ± 1.48^b^
NaCl	2555.81 ± 116.90^a^	926.13 ± 90.56^c^	2.78 ± 0.31^a^	667.96 ± 64.52^c^	79.25 ± 7.86^d^	8.48 ± 1.09^a^
H_2_S+ NaCl	3541.94 ± 256.95^b^	667.26 ± 65.89^a^	5.35 ± 0.73^cd^	787.98 ± 43.05^d^	32.81 ± 5.83^b^	24.42 ± 3.48^d^
H_2_S+ HT+ NaCl	2456.45 ± 172.61^a^	625.76 ± 48.76^a^	3.95 ± 0.48^b^	687.81 ± 12.85^c^	39.75 ± 4.26^c^	17.43 ± 1.87^c^

*Control, H_*2*_S, H_*2*_S + HT, NaCl, H_*2*_S + NaCl, H_*2*_S + HT + NaCl correspond to the group of seedlings exposed to only nutrients, 50 μM NaHS, 50 μM NaHS + 500 μM hypotaurine, 150 mM NaCl, 50 μM NaHS + 150 mM NaCl and 50 μM NaHS + 500 μM hypotaurine + 150 mM NaCl, respectively. FW, fresh weight; DHA, dehydroascorbic acid; GSSG, oxidized glutathione. Values are means ± SD of three independent replications (*n* = 3). Different superscripted letters (a, b, c, and d) within the column indicate statistically significant differences among the treatments according to Duncan’s multiple range test (*P <* 0.05)*.

### H_2_S Stimulates the Activities of Various Antioxidant Enzymes

**FIGURE 4:**
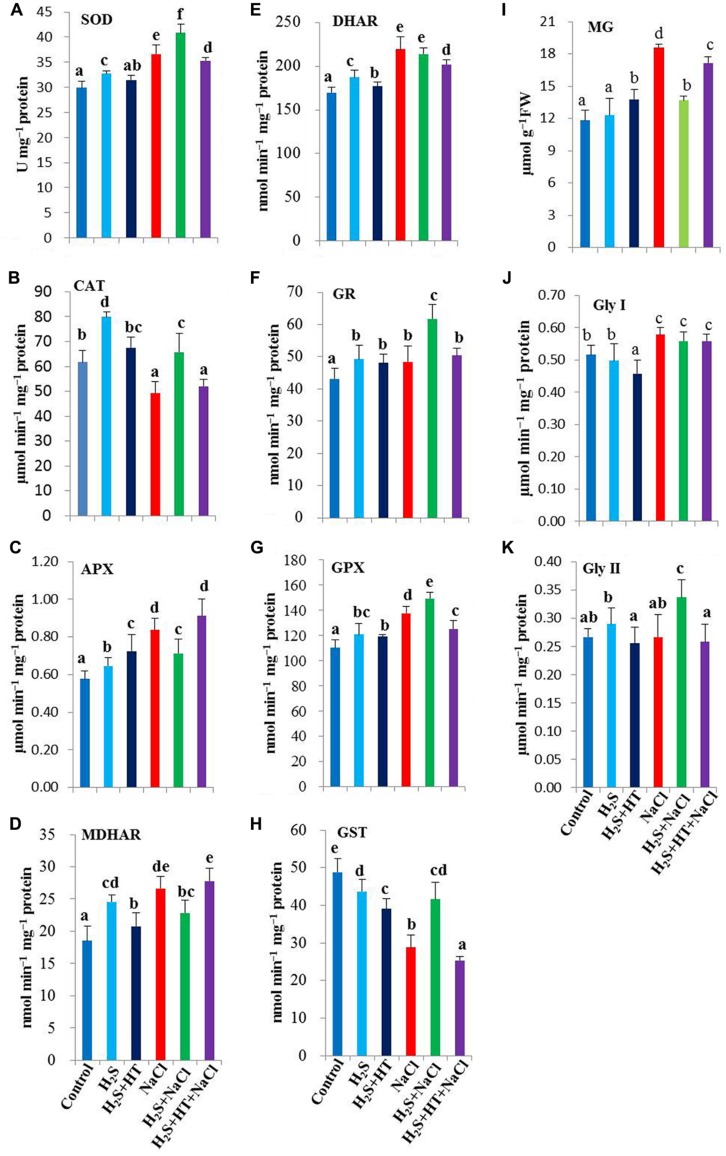
**Effect of H_2_S on ROS and MG detoxification systems in the leaves of salt-stressed rice plants pretreated with or without NaHS and HT**. Activities of ROS detoxifying enzymes **(A)** superoxide dismutase (SOD), **(B)** catalase (CAT), **(C)** ascorbate peroxidase (APX), **(D)** monodehydroascorbate reductase (MDHAR), **(E)** dehydroascorbate reductase (DHAR), **(F)** glutathione reductase (GR), **(G)** glutathione peroxidase (GPX) and **(H)** glutathione *S*-transferase (GST). **(I)** methylglyoxal (MG) content, **(J)** glyoxalase (Gly) I and **(K)** Gly II activities. Control, H_2_S, H_2_S + HT, NaCl, H_2_S + NaCl, H_2_S + HT + NaCl correspond to the group of seedlings subjected to only nutrients, 50 μM NaHS, 50 μM NaHS + 500 μM HT, 150 mM NaCl, 50 μM NaHS + 150 mM NaCl and 50 μM NaHS + 500 μM HT + 150 mM NaCl, respectively. Bars represent standard deviation (SD) of the mean (*n* = 3). Different letters (a, b, c, d, e, and f) indicate significant differences among the treatments at *P <* 0.05, according to Duncan’s multiple range test. FW, fresh weight; HT, hypotaurine.

In our subsequent experiments, we evaluated the regulatory role of H_2_S on the activities of various antioxidant enzymes involved in ROS metabolism. Experimental results revealed that NaCl treatment displayed an increase in SOD activity by 22% and a sharp decline in CAT activity by 20%, as compared with NaCl-free control (**Figures 4A,B**). NaHS pretreatment further enhanced the SOD activity, while rescuing CAT activity, but both of which were differentially inhibited when the rice plants were co-treated with NaHS and HT prior to salt stress treatment. H_2_S-induced elevation of SOD and CAT activities corroborated with the levels of $O_{2}^{\cdot -}$ and H_2_O_2_ in the leaves of rice plants under salt stress (**Figures 3A,B and 4A,B**). Interestingly, under NaCl-free conditions, NaHS pretreatment significantly enhanced the activities of SOD and CAT compared with control.

The synergistic actions of the enzymes of ascorbate-glutathione cycle eliminate H_2_O_2_ using AsA and GSH as co-factors (Gill and Tuteja, 2010). Our results showed a considerable increase in the activity of APX in salt-stressed alone seedlings, with 22.4% higher than the untreated control (**Figure 4C**). We also observed that salt stress alone significantly enhanced the activities of AsA- and GSH-regenerating enzymes, including MDHAR, DHAR and GR, by 44, 29, and 12%, respectively (**Figures 4D–F**). Although pretreatment with NaHS did not show boosting effects on APX, MDHAR and DHAR activities under salt stress, their activity remained significantly higher than that of control. On the other hand, GR activity was further enhanced by 28% upon NaHS pretreatment of salt-stressed plants compared with that in salt-stressed only plants (**Figure 4F**). By contrast, co-application of NaHS and HT prior to salt exposure displayed a trend of these enzyme activities similar to salt stressed only seedlings. Notably, under NaCl-free conditions, NaHS and NaHS + HT pretreatments significantly increased the activities of these four enzymes, as compared with untreated control.

We further evaluated the effects of H_2_S on the activities of GSH-metabolizing enzymes, such as GPX and GST. Our results showed differential responses of GPX and GST under salt stress, with GPX activity being increased by 25%, whereas GST activity being drastically decreased by 41% in comparison with untreated control (**Figures 4G,H**). The inducible tendency of GPX activity was further strengthened by NaHS pretreatment, but reversed to a lower level by NaHS and HT co-treatment. In case of GST, NaHS pretreatment significantly rescued the activity of GST, while NaHS + HT pretreatment further aggravated its decreasing tendency upon NaCl stress exposure. Following NaHS and NaHS + HT treatments, GPX activity showed significant enhancements but GST activity decreased by 11 and 20%, respectively, in unstressed plants as compared with control (**Figures 4G,H**).

### H_2_S Upregulates MG-detoxifying Gly System

The cytotoxic aldehyde MG is detoxified by the maintenance of GSH homeostasis via Gly enzymes (Yadav et al., 2005; Upadhyaya et al., 2011). NaCl treatment resulted in a noticeable increase of MG level by 58%, as compared with control (**Figure 4I**). A significant inhibition of MG production was observed in NaHS-pretreated salt-stressed plants when compared with the plants stressed with salt alone. Moreover, NaCl-triggered production of MG was sustained in NaHS + HT-pretreated salt-stressed plants. In the presence or absence of NaHS and HT, MG-detoxifying enzymes exhibited differential responses in salt-stressed seedlings (**Figures 4J,K**). Salt stress increased Gly I activity by 12%, but did not alter the Gly II activity as compared with the untreated control. On the other hand, NaHS and NaHS + HT pretreatments prior to salt stress kept the Gly I activity at the level of NaCl-stressed only plants. However, NaHS pretreatment showed significant increase in the activity of Gly II in salt-stressed plants when compared with salt-stressed only plants. By contrast, adding H_2_S scavenger HT along with NaHS showed a decrease in Gly II activity in salt-stressed seedlings (**Figure 4K**). Thus, upregulation of Gly enzymes by administration of NaHS might contribute to the reduction of NaCl-induced MG toxicity in rice plants.

### H_2_S Improves Phenotypes of Rice Plants

**FIGURE 5:**
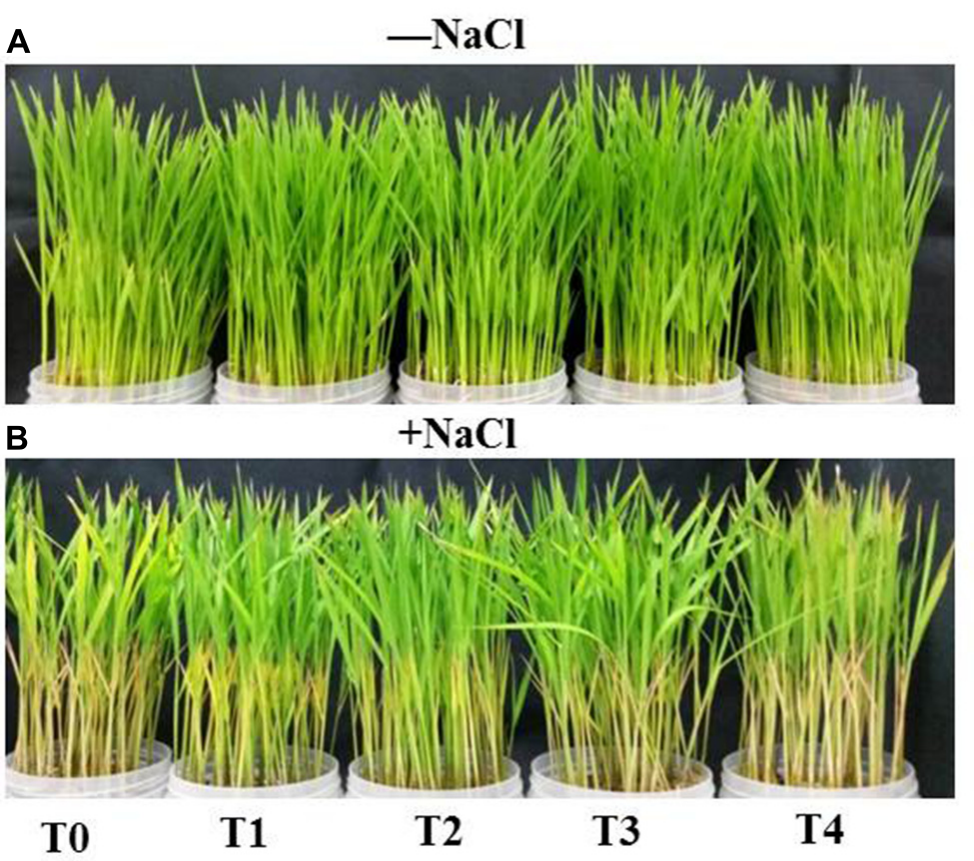
**Effects of H_2_S on phenotypes of rice plants in the absence **(A)** and presence **(B)** of salt (NaCl)**. Twelve-day-old plants were pretreated with 0, 25 μM H_2_S, 50 μM H_2_S, 100 μM H_2_S and 50 μM H_2_S + 500 μM HT for 48 h and then subjected to 150 mM NaCl stress for 4 days. Photographs of the plants were taken after 4 days of NaCl treatment. T0, T1, T2, T3, and T4 correspond to 0 μM NaHS, 25 μM NaHS, 50 μM NaHS, 100 μM NaHS and 50 μM NaHS + 500 μM HT, respectively. HT, hypotaurine.

As shown in **Figure 5** salt treatment of rice plants for 4 days exhibited toxicity symptoms, which were manifested as severe yellowing, chlorosis, burning and roiling of the leaves of rice plants (**Figure 5B**). Salt stress also induced stunted growth, as observed by reduction in plant height (**Figure 2D**). In comparison with salt-stressed only seedlings, NaHS-pretreated rice plants showed considerably less toxicity upon salt treatment. Among the doses of NaHS, 50 μM NaHS was best in terms of the physiological and morphological observations. Contrary, co-pretreatment with NaHS and HT further aggravated salt-induced toxicity symptoms. Under non-stress conditions, treatment of rice plants with NaHS or NaHS + HT for 48 h did not exert any toxicity symptoms (**Figure 5A**).

## Discussion

**FIGURE 6:**
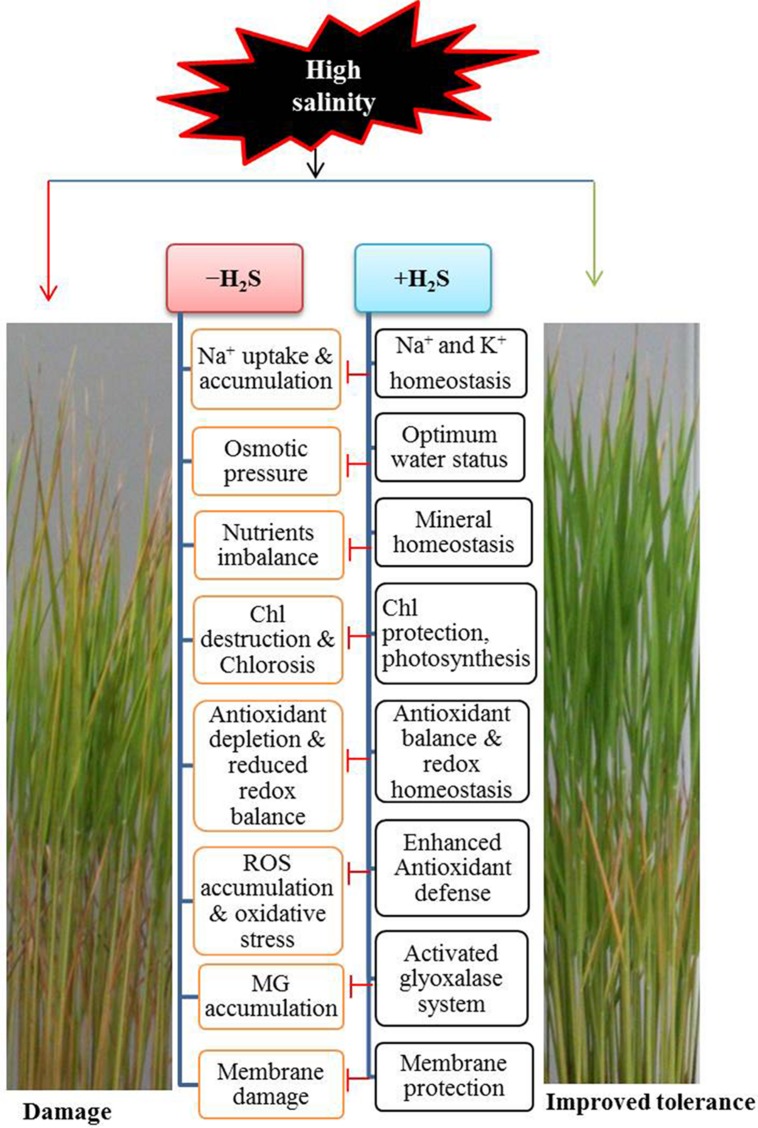
**A possible model showing the mechanism of H_2_S-induced rice tolerance to salt stress**. Exposure of rice plants to NaCl caused an increased in uptake and accumulation of Na^+^ in plant cells. This increased level of toxic ions is responsible for inducing osmotic stress, chlorosis, reactive oxygen species (ROS) and methylglyoxal (MG) accumulation, membrane damage and ionic disorder, all of which significantly affect the plant health, leading to cell death and damage of plants. On the other hand, H_2_S supplementation resulted in Na^+^ and K^+^ homeostasis, osmoregulation, increased photosynthesis, higher antioxidant capacity, enhanced MG detoxification, membrane stabilization and ion balance, all of which contributed to the alleviation of salt-induced damage, leading to improved tolerance to salt stress. Chl, chlorophyll; Blunted arrow indicates inhibitory effects.

Plant tolerance to salinity is a genetic trait that differs greatly among species, as reflected in their growth responses under certain conditions. Of the various cereal crops, barley is the most tolerant and rice is the most sensitive to salt stress (Munns and Tester, 2008). Therefore, a slight accumulation of Na^+^ and Cl^-^ in cells may adversely affect rice growth and development by impairing various physiological and biochemical processes, including membrane permeability, mineral homoeostases, photosynthesis, stomatal aperture, redox homeostasis, and ROS production and detoxification (**Figure 6**) (Munns and Tester, 2008; Gupta and Huang, 2014; Parihar et al., 2015). Consequently, plants have evolved sophisticated mechanisms to flexibly adapt themselves to overcome salinity-induced damage. However, under stressful conditions, plants’ basal capacity overwhelms and plants need to boost the protective mechanisms in order to surmount the detrimental consequences. Our investigations clearly showed that the promotion of H_2_S level alleviated, and reduction of its level aggravated salt-induced toxic effects, which were clearly observed in the levels of H_2_O_2_ and MDA as well as in the phenotypes of rice plants (**Figures 1A,B**, **Figures 3A–E**, **5** and **6**). The benefit of modulation of endogenous H_2_S has also been observed in other plant species like *Zea mays* under heat, *Arabidopsis thaliana* under drought and *Medicago sativa* under salt stress conditions (Jin et al., 2011; Li et al., 2013; Lai et al., 2014). Our study provided firm evidence that H_2_S considerably prevented Na^+^ uptake, whereas increasing the uptake of K^+^, thereby restraining the increased Na^+^/K^+^ ratio (**Figures 2A–C** and **4**). This might be achieved through the maintenance of plasma membrane integrity, as was observed in *Arabidopsis* and alfalfa (Li et al., 2013; Lai et al., 2014). Furthermore, NaHS enhanced the contents of essential minerals under salt stress (**Table 1**) that are essential for activating a number of physiological and biochemical events associated with stress adaptations. One of the initial effects of high salinity is the reduction of plant growth, which is accompanied by salt-induced osmotic stress (Munns and Tester, 2008). Our results implied that H_2_S improved overall growth and biomass of salt-stressed rice plants which could be attributed to its role in protecting Chl a, Chl b, carotenoids and proteins from salt-induced damage (**Table 2**, **Figures 2D–F** and **6**). Moreover, H_2_S might have contributed to improving photosynthetic performance, and thus overall performance of rice plants by delivering Mg, Fe, Zn, and Mn ions as well as by promoting biogenesis of chloroplast and increasing the ability of CO_2_ fixation, as observed in *Spinacea oleracea* (Chen et al., 2011). Under osmotic stress, accumulation of compatible solutes like Pro is a universal response plants adapted to retain water status (Hayat et al., 2012). However, Pro accumulation is not always correlated with stress tolerance, which rather depends on the turnover of Pro metabolism (Kishor and Sreenivasulu, 2014). Several studies have claimed that the level of accumulated Pro reflects the intensity of stressed symptoms when plants were challenged with different types of abiotic stresses (Di Martino et al., 2003; Metwally et al., 2003; Loutfy et al., 2012; Mostofa et al., 2015c) as also observed in this study. Similarly, Chang et al. (2014) recently reported that Pro accumulation in salt-stressed *Cathranthus roseus* negatively correlated with RWC, biomass and K^+^ accumulation. We assumed that H_2_S pretreatment reduced accumulation of Na^+^, and as a consequence reduced water loss rate; thus, high level of Pro synthesis might be unnecessary (**Table 2**). Consistent with our results, Li-xu et al. (2013) observed that NaHS application reduced salt-induced accumulation of Pro in the hypocotyls and radicles of cucumber seedlings.

Salt stress-induced water deficit and -increased ionic and osmotic effects often lead to the formation of excessive ROS that can generate oxidative stress by oxidizing lipid, proteins and nucleic acids (Miller et al., 2010; Bose et al., 2014). As expected, high salinity-triggered overproduction of $O_{2}^{\cdot -}$ and H_2_O_2_, and increased LOX activity in leaf tissues were correlated with the significant increase in MDA level (**Figures 3A–E**). This detrimental effect was also coincided with the impaired ROS detoxifying capacity, as evident by dropped level of AsA and imbalance of the redox states in terms of reduced AsA/DHA and GSH/GSSG ratios (**Table 3**). Furthermore, unsynchronized activities of SOD and CAT (**Figures 4A,B**), which constitute the frontline enzymatic network by converting $O_{2}^{\cdot -}$ and H_2_O_2_ consecutively into H_2_O, accelerated the accumulation of ROS in salt-stressed rice plants (**Figures 3A,B**). On the other hand, H_2_S successfully alleviated salt-induced oxidative damage, as evident by reduced accumulation of $O_{2}^{\cdot -}$ and H_2_O_2_, as well as lipid peroxidation product MDA in rice leaves (**Figures 3A–E** and **6**). H_2_S executed its antioxidant role, mainly by increasing the levels of AsA and GSH, and by reestablishing redox homeostasis (**Table 3**). Moreover, enhancement of SOD and CAT activities also indicated that H_2_S kept a balance between ROS production and detoxification, thereby protecting cells from oxidative damage (**Figure 6**). The enzymes APX, MDHAR, DHAR and GR of the ascorbate-glutathione cycle act coordinately in eliminating H_2_O_2_ and regenerating AsA and GSH, which is crucial for promoting plant tolerance to abiotic stresses (Gill and Tuteja, 2010; Miller et al., 2010). In consistence with the findings of Mishra et al. (2013), this study observed an accumulation of H_2_O_2_ even after increasing the activities of these four enzymes, suggesting that production of H_2_O_2_ exceeded ROS-scavenging capacity in salt stressed plants (**Figures 3B,C** and **4C–F**). In contrast, H_2_S regulates the ascorbate-glutathione cycle differentially by keeping APX, MDHAR and DHAR activities above the control level, while further intensifying GR activity (**Figures 4C–F**). We assumed that coordinating role of SOD and CAT exerted reduced oxidative load on ascorbate-glutathione cycle, resulting in less stimulation of the activities of these enzymes. Furthermore, H_2_S-induced enhancement of GR activity contributed well to maintaining redox status, presumably by regenerating GSH from GSSG, which corroborated with the observed increase in GSH level and GSH/GSSG ratio (**Table 3**, **Figure 4F**). It is well known that GSH-dependent defense mechanisms play significant role in protecting plants against various stresses, including salt stress (Mittova et al., 2003; Anjum et al., 2014; Nazar et al., 2014). Besides scavenging ROS directly, GSH detoxifies a wide variety of lipid hydroperoxides, reactive aldehydes and endobiotic substrates with the help of GPX and GST (Mittova et al., 2003). A careful analysis of GSH-related defense mechanism under H_2_S addition revealed that H_2_S further enhanced GSH level, possibly by inducing its biosynthesis or by upregulating GR activity under salt stress, which then participates in redox homeostasis, as well as GPX and GST-mediated efficient scavenging of peroxides and other electrophiles (**Figures 4G,H**). Similar to our findings, Dawood et al. (2012) and Li-xu et al. (2013) also reported that H_2_S-induced GPX and GST activities contributed to enhanced tolerance to salt and aluminum stresses in *Cucumis sativus* and *Hordeum vulgare*, respectively.

Additionally, we investigated the effects of H_2_S on another GSH-dependent defense mechanism, namely Gly system, which is involved in MG detoxification and redox balance (Upadhyaya et al., 2011; Hossain et al., 2012; Alvarez Viveros et al., 2013). We noticed that salt stress resulted in a significant increase in MG content, which might be due to the inefficient and desynchronizing activities of Gly enzymes, as Gly I activity increased but Gly II activity remained at the control level (**Figures 4I–K**). Moreover, an inefficient Gly II activity might also trap GSH by accumulating *S*-lactoylglutathione (SLG), which is also mutagenic and cytotoxic (Hossain et al., 2012). However, H_2_S addition exhibited an encouraging result on MG detoxification, as evident by a correlation between decreased MG content and increased activities of Gly enzymes, which ultimately protected the cells form MG and SLG toxicity. Furthermore, our result also assumed that enhanced Gly II activity efficiently recycled GSH into the system, which eventually facilitated GSH homeostasis and higher activities of GPX and GST (**Table 3**, **Figures 4G,H,J**) in preventing oxidative stress (**Figures 3A–E** and **6**). These results are consistent with previous reports (Mustafiz et al., 2011; Upadhyaya et al., 2011; Lakra et al., 2015; Mostofa et al., 2015a,b) and suggest that H_2_S coordinates the biochemical actions of the antioxidant defense and Gly systems by maintaining GSH level to mitigate the salt-induced ROS and MG toxicity in rice plants.

In summary, we conclude (**Figure 6**) that modulating the level of endogenous H_2_S could be an important strategy in improving performance of rice plants under salt stress for the following reasons. First, H_2_S restricts the uptake of Na^+^, thereby maintaining the Na^+^/K^+^ ratio. Second, H_2_S maintains mineral homeostases, which smoothens minerals driven physiological and biochemical processes. Third, H_2_S enhances photosynthetic capacity by protecting photosynthetic pigments. Fourth, H_2_S reduces oxidative damage, principally by regulating the GSH-based antioxidant defense and MG detoxification systems. Thus, our findings provide a solid foundation for developing salt-tolerant rice and perhaps other crops by fine-tuning the level of endogenous H_2_S through genetic engineering of H_2_S biosynthesis.

## Author Contributions

MM conceived, designed, and conducted the experiments. DS helped in conducting experiments. MM and L-SPT analyzed the data and results. MM and L-SPT wrote the manuscript. MF monitored the experimental work and critically commented on the manuscript. All authors read and approved the final manuscript.

## Conflict of Interest Statement

The authors declare that the research was conducted in the absence of any commercial or financial relationships that could be construed as a potential conflict of interest.
